# *Streptococcus agalactiae* Serotype IV in Humans and Cattle, Northern Europe^1^

**DOI:** 10.3201/eid2212.151447

**Published:** 2016-12

**Authors:** Ulrike Lyhs, Laura Kulkas, Jørgen Katholm, Karin Persson Waller, Kerttu Saha, Richard J. Tomusk, Ruth N. Zadoks

**Affiliations:** University of Helsinki, Seinäjoki, Finland (U. Lyhs); Valio Ltd., Helsinki, Finland (L. Kulkas);; SEGES (formerly Knowledge Centre for Agriculture), Aarhus, Denmark (J. Katholm);; National Veterinary Institute, Uppsala, Sweden (K. Persson Waller); Central Hospital, Seinäjoki (K. Saha);; University of Glasgow, Glasgow, Scotland, UK (R.J. Tomusk, R.N. Zadoks);; Moredun Research Institute, Penicuik, Scotland, UK (R.N. Zadoks)

**Keywords:** Streptococcus agalactiae, human, cattle, bovine mastitis, molecular epidemiology, host specificity, zoonoses, bacteria, Europe

## Abstract

*Streptococcus agalactiae* is an emerging pathogen of nonpregnant human adults worldwide and a reemerging pathogen of dairy cattle in parts of Europe. To learn more about interspecies transmission of this bacterium, we compared contemporaneously collected isolates from humans and cattle in Finland and Sweden. Multilocus sequence typing identified 5 sequence types (STs) (ST1, 8, 12, 23, and 196) shared across the 2 host species, suggesting possible interspecies transmission. More than 54% of the isolates belonged to those STs. Molecular serotyping and pilus island typing of those isolates did not differentiate between populations isolated from different host species. Isolates from humans and cattle differed in lactose fermentation, which is encoded on the accessory genome and represents an adaptation to the bovine mammary gland. Serotype IV-ST196 isolates were obtained from multiple dairy herds in both countries. Cattle may constitute a previously unknown reservoir of this strain.

## Abstract 2

Cattle may be a reservoir for this emerging pathogen of humans.

*Streptococcus agalactiae* (group B *Streptococcus*) is a major cause of neonatal infectious disease in humans in many countries and is carried asymptomatically by a large proportion of adults. It is also recognized as an emerging pathogen in human adults worldwide and as a reemerging mammary pathogen of cattle in northern Europe (*1**–**3*). In adults, *S. agalactiae* is primarily associated with bacteremia, skin and soft tissue infections (SSTI), and urinary tract infections (UTI) and occasionally with necrotizing fasciitis, arthritis, toxic shock syndrome, endocarditis, or meningitis (*4**–**6*). Host and pathogen factors contribute to the emergence of *S. agalactiae* among adults (*3*). Among hosts, at greater risk are elderly patients and persons with chronic underlying conditions such as alcohol abuse, diabetes mellitus, or immunosuppression (*2**,**4**,**6*). Within the pathogen, new strains such as serotype IV may contribute to disease emergence (*7**,**8*). Considering the risk factors for *S. agalactiae* in nonpregnant adults and demographic changes in many countries, the incidence of group B streptococcal disease can be anticipated to increase (*4*).

In the 1950s, *S. agalactiae* was the most common mastitis-causing bacterium among dairy cattle in Europe, severely reducing milk quality and quantity. In the 1960s, development of disease control programs and introduction of legislation resulted in near eradication of *S. agalactiae* from several northern European countries, a situation that continued until the end of the 20th century (*9*). In the 21st century, farm management in northern Europe changed (e.g., fewer herds, increased average herd size, and introduction of automated milking systems). Concomitantly, the prevalence of *S. agalactiae* in bovine milk increased. In Denmark, in the first years of the 21st century, the proportion of *S. agalactiae*–positive herds tripled (*1**,**9*). Similar phenomena have been described for Sweden and Norway (*1*).

The presence of *S. agalactiae* in humans and cattle raises the question of whether interspecies transmission occurs. This question is particularly pertinent in light of the emergence of *S. agalactiae* disease in adult humans and its reemergence in cattle. Several comparisons of *S. agalactiae* in humans and cattle have been published, and most authors conclude that isolates from these species form largely distinct populations with regard to the core genome and the accessory genome (*10**–**12*). Ideally, assessment of the potential for interspecies transmission is based on the analysis of contemporaneous, sympatric isolates. With 1 exception (*13*), however, most comparative studies were not based on isolates from the same geographic region and the same period, or, if they were, they covered a very limited number of *S. agalactiae–*positive farms or animals (*14**,**15*). Our aim with this study was to provide insight into the hazard of interspecies transmission of *S. agalactiae* by comparing contemporaneous populations of *S. agalactiae* from humans and dairy cattle in Finland and Sweden. 

## Materials and Methods

### Isolates

A total of 81 isolates were collected at the Seinäjoki Central Hospital in the rural South Ostrobothnia district of Finland (2011, 2012) and from epidemiologically unrelated persons by the Department of Clinical Microbiology, University Hospital, Uppsala, in an urban area of Sweden (2012, 2013). Isolates originated from 12 patients with invasive disease (sepsis or meningitis), 37 with UTI, and 15 with SSTI and from 17 healthy carriers who were screened during pregnancy by use of vaginal swab or cervical fluid samples. Isolates represented a convenience sample that covered both sexes and all age classes (Technical Appendix Figure 1). Data were not collected with regard to farm or animal contact or dietary exposure.

During 2010–2012, a total of 108 isolates from cattle were collected by the laboratory of Valio Ltd, Helsinki, Finland (63 isolates from 29 herds) and by the National Veterinary Institute, Uppsala, Sweden (45 isolates from 45 herds). Isolates were cultured from individual cow or quarter milk samples from animals with suspected intramammary infection with or without clinical signs. In both countries, samples originated from most of the major dairy regions (Technical Appendix Figure 2). For 9 herds in Finland, isolates from multiple cows were available and 2–14 isolates/herd were used to assess within-herd strain heterogeneity (Technical Appendix Table 1). For the remaining herds, 1 isolate was used.

Phenotypic identification was based on colony morphology on blood agar, CAMP (Christie, Atkins, Munch-Peterson) reaction, and Lancefield grouping (*15*). Before use, isolates were stored at −80°C in brain–heart infusion broth (Oxoid, Basingstoke, UK) with 15% glycerol. After culture on blood agar, purity was checked and 1 colony was used to confirm species identity by PCR with primers STRA-AgI (5′-AAGGAAACCTGCCATTTG-3′) and 5′-STRA-AgII (TTAACCTAGTTTCTTTAAAACTAGAA-3′). DNA extracts for molecular typing were prepared from overnight cultures in brain–heart infusion broth by using a DNeasy Blood & Tissue Kit (QIAGEN, Manchester, UK). Species confirmation, multilocus sequence typing (MLST), and serotyping were conducted for all isolates. Pilus island (PI) typing and lactose typing were conducted for all isolates from humans and 1 isolate from a bovid per sequence type (ST) per herd.

### MLST

MLST was performed by using standard primers and HiMLST or Sanger sequencing of purified PCR amplicons (*16**,**17**)*. Alleles and STs were assigned by using the *S. agalactiae* database (http://pubmlst.org/sagalactiae/) (*18*). New alleles were submitted to the database curator for quality control and allocation of allele numbers and STs. Novel allele combinations were also submitted for ST assignment.

### Molecular Serotyping

For detection of molecular serotype (MS) II and MS IV, duplex PCR was used, and for detection of MS V, VI, VII, and VIII, multiplex PCR was used (*19*). PCR reactions for MS Ia, Ib, and III were run individually by using primers for MS Ia and Ib (*19*) and primers IIIcpsHS and IIIcpsHA for MS III (*20*). For all reactions, cycling conditions were 94°C (5 min), followed by 40 cycles of 94°C (60 s), 55°C (60 s), and 72°C (60 s) with final extension at 72°C (5 min). Each isolate was submitted to all molecular serotyping reactions to identify potential cross-reactivity.

### PI Typing

Multiplex PCR was used to screen for presence of *sag647* (PI-1), s*ag1406* (PI-2a), and *san1517* (PI-2b); the housekeeping gene *adhP* was used as amplification control (*11*). Isolates that were negative for PI-1 were tested for presence of an intact integration site. Detection of a 684-bp amplicon indicates presence of an intact site, and absence of the amplicon indicates occupation by an alternative, uncharacterized genetic element. Isolates that were positive for PI-2a or PI-2b were further characterized by PI-specific PCR-based restriction fragment length polymorphism analysis to detect allelic variation in the PI-2a adhesin gene (*gbs59*) and the PI-2b backbone protein (*san1519)* (*11*).

### Lactose Typing

To detect lactose fermentation, we inoculated a single colony into phenol red broth (neutralized soya peptone with beef extract; Oxoid), supplemented with phenol red and α lactose (L2643; Sigma-Aldrich, Gillingham, UK). Broth was incubated at 37°C without shaking and was checked for change from red to yellow at 24 h, 48 h, and 7 days after inoculation. PCR was used to screen for presence of an ≈2.5-kbp region of *lacEFG*, which is part of the Lac.2 operon that encodes lactose fermentation (*21*).

### Data Analysis

Comparisons of categorical variables were conducted in Statistix 10 (Analytical Software, Tallahassee, FL, USA) with use of the Fisher exact or Pearson χ^2^ test, as appropriate. Global eBURST analysis was performed by using PHYLOViZ (*22*); double-locus variants were included in eBURST groups.

## Results

### MLST

All isolates were confirmed as *S. agalactiae* and 33 STs were identified. Isolates from humans belonged to 16 allelic profiles, including 2 new profiles derived from isolates from patients with invasive disease in Sweden. Both profiles were single-locus variants (SLVs) of known STs, with ST751 based on a new combination of known alleles, whereas the second profile was based on a new *atr* allele with an internal deletion (ST not assigned). ST1 was most common, followed by ST19 and ST12 (21, 14, and 10 isolates, respectively). All STs were found in isolates from patients in at least 2 age groups and from 2 clinical sample types (Technical Appendix Figure 3).

Among 108 isolates from cattle, 22 STs were identified, including 12 new STs (5 from Finland, 7 from Sweden). The proportion of new STs was higher among isolates from cattle than from humans (54.5% vs. 12.5%, respectively; Pearson χ^2^ = 4.7, df = 1, p = 0.03). A total of 3 new STs (ST632, ST633, ST726) were detected in multiple herds, whereas the remaining new STs (ST634–636, ST722–725, ST727, ST728) were each obtained from 1 herd. All were SLVs of known STs with 1 new allele. For 9 herds, >1 isolate was available and isolates within a herd belonged to a single ST, with 1 exception in which ST1 and its SLV ST635 were detected (Technical Appendix Table 1). Both isolates from this herd were included in herd-level analysis and comparison between host species. In herd-level analysis of 74 isolates, ST1 was most common, followed by ST103 and ST196 (20, 10, and 8 herds, respectively; Technical Appendix Table 2). We found no significant association between ST and country of origin.

**Figure 1:**
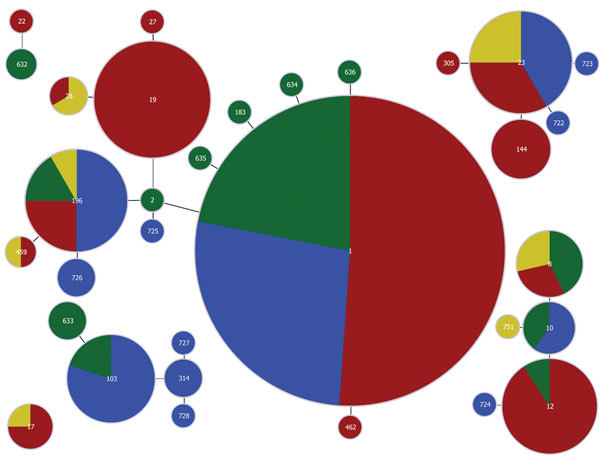
Distribution of host species and countries across clusters of *Streptococcus agalactiae* sequence types (STs), with clusters including single- and double-locus variants. Each circle represents an ST, with size of the circle and its colored segments proportional to the number and origin of isolates, respectively. Red, human in Finland; yellow, human in Sweden; green, bovid in Finland; blue, bovid in Sweden. STs are indicated by numbers in the circles. Single- and double-locus variants are connected by black lines.

Of 33 STs in this study, 5 were detected in both host species (Figure 1). ST1 was the most common shared ST, followed by ST23, ST196, ST12, and ST8 (41, 12, 12, 11, and 7 isolates, respectively). More than half (84) of the 155 isolates (54.2%) belonged to shared STs. Of 5 shared STs, 4 were represented by >10 isolates compared with 2 of 28 host-specific STs (p<0.001 by Fisher exact test). Using global eBURST, we identified 6 clusters, 2 of which were host specific (i.e., ST17 from humans and a cluster around ST103 from cattle). Both host-specific clusters included isolates from both countries (Figure 1).

### Molecular Serotyping

Among isolates from humans, MS Ia, Ib, and II–VI were identified. Among isolates from cattle, 2 did not yield conclusive molecular serotyping results. Among the remaining isolates from cattle, MS Ia, Ib, II–V, and VII were identified. We found strong correlation between MS and ST (Table; Technical Appendix Figure 4). For STs found in both host species, MS did not differ between isolates from humans or cattle, with the exception of ST23: isolates from humans belonged to MS Ia and those from cattle to MS Ia or III (Table).

**Table:** *Streptococcus agalactiae* isolates from humans and cattle, Finland and Sweden, 2010–2013*

Molecular serotype	No. isolates from humans/cattle							No. isolates from humans only					No. isolates from cattle only				
	ST1	ST8	ST12	ST23	ST196	Other		ST17	ST19	ST28	ST144		ST10	ST103	ST314	ST633	ST726
Ia	1/0	–	–	7/2	–	1/3		–	–	–	6		–	10	3	3	–
Ib	–	4/3	–	–	–	1/0		–	–	–	–		1	–	–	–	–
II	1/0	–	10/1	–	–	2/1		–	1	3	–		3	–	–	–	–
III	–	–	–	0/2	–	1/4		4	13	–	–		–	–	–	–	–
IV	–	–	–	–	4/8	2/1		–	–	–	–		–	–	–	–	3
V	18/19	–	–	–	–	1/4		–	–	–	–		–	–	–	–	–
Other	1/1	–	–	0/1	–	–		–	–	–	–		1	–	–	–	–

*ST, sequence type; –, no isolates.

### PI Typing

**Figure 2:**
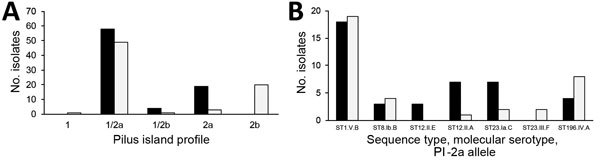
Distribution of pilus island profiles (A) and alleles within pilus island 2a (B) across *Streptococcus agalactiae* isolates from humans (dark bars) and bovids (light bars). Letter and number combinations in B show sequence type (ST), molecular serotype (Roman numeral), and allele for pilus island 2a (capital letter).

We identified 5 PI profiles (Figure 2; Technical Appendix Figure 5). Among PI-1–negative isolates, the integration site was intact in 14 of 19 isolates from humans and in 8 of 23 isolates from cattle (74% vs. 35%, χ^2^ = 6.31, df = 1, p = 0.01). Within host–ST combinations, results for PI-1 and occupation of the integration site were consistent across isolates (Technical Appendix Table 2). Across both host species, 1 PI-2a allele was identified in ST1, ST8, and ST196, respectively. ST12 included 2 PI-2a alleles among isolates from humans (Figure 2). Within ST23, PI-2a alleles were MS specific. One combination was identified in both host species, and 1 was limited to cattle (Figure 2). PI-2b and PI-1 were present in all ST17 isolates (from humans) and 1 ST724 isolate (from cattle), and PI-2b alone was present in isolates with cattle-specific STs (Technical Appendix Figure 5). One PI-2b allele was found in ST632, and a second allele was found across the entire bovine-specific eBURST cluster.

### Lactose Typing

**Figure 3:**
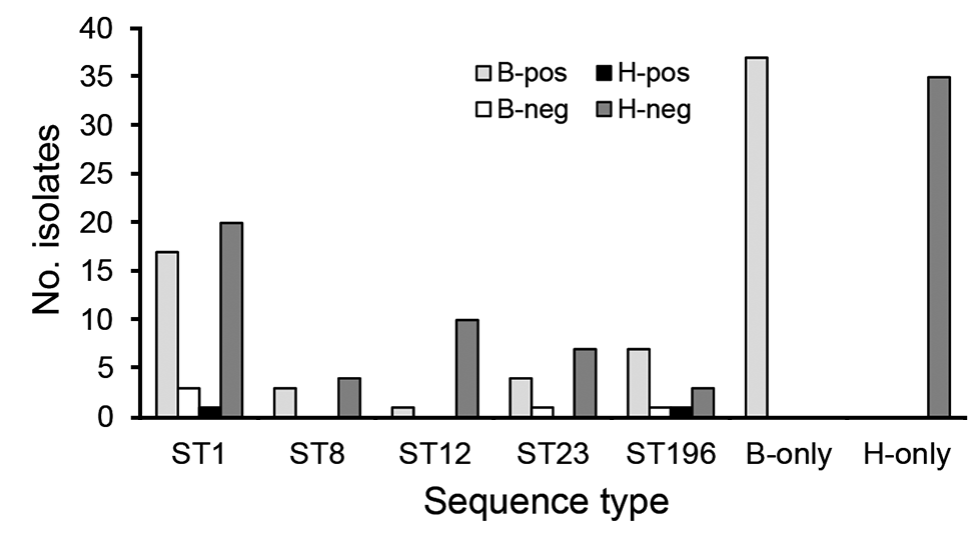
Distribution of *lacEFG* PCR–positive (pos) and –negative (neg) human (H) and bovine (B) *Streptococcus agalactiae* isolates across sequence types (ST). STs found in both host species are shown individually, whereas STs that were found in a single species are grouped by species.

Of 81 isolates from humans and 73 isolates from cattle, lactose was fermented by 3 (3.7%) and 73 (100%), respectively (χ^2^ = 142, df = 1, p<0.001). One isolate from cattle was nonviable and hence not tested. Of 81 isolates from humans, 2 (2.5%) were positive for *lacEFG* compared with 69 (94.5%) of 73 isolates from cattle (χ^2^ = 131, df = 1, p<0.0001). Discrepancies between phenotype and genotype were confirmed by repeating culture, DNA extraction, and phenotypic and genotypic testing. Genotypic results that were atypical for the host species were observed only in STs that were found in both host species (i.e., in ST1, ST23, and ST196) (Figure 3).

## Discussion

In contrast to results of previous studies (*11**,**12*), our results showed no clear distinction between subpopulations of *S. agalactiae* from humans or cattle according to MLST, molecular serotyping, or PI typing. With few exceptions (*13**,**14*), previous comparative studies were not based on contemporaneous, sympatric isolates across host species. Our study showed that ≈54% of isolates belonged to a population that affects both host species. The convenience sample used may not be fully representative of the distribution of STs across the human population in those countries, and invasive isolates were obtained only from Sweden, but no significant clustering by country or clinical manifestation was observed, suggesting that the observed ST distribution is broadly indicative of the most common types.

The most prevalent shared ST was ST1. Its presence in cattle from several countries has been described, but high prevalence among cattle has been reported in Denmark only (*11**,**12**,**23*). Serotype V and PI type 1/2a were predominant in ST1 from both host species, a finding that agrees with findings from previous studies (*11**,**24*). Surprisingly, ST196 was the second most common shared ST (equally with ST23), and all carried MS IV. Reports of ST196 and serotype IV used to be rare; only 1 of 158 isolates collected in Sweden over a 10-year period (1988–1997) belonged to ST196, but they are now recognized as emerging human pathogens (*7**,**8**,**25*). Furthermore, emergence of new STs with MS IV in humans, such as ST459, has been described (*7**,**8*). We describe the emergence of a new MS IV strain in cattle, ST723, in multiple dairy herds in Sweden. The relatively common occurrence of ST196 and its SLVs in cattle in the Nordic countries (this study; *26*) suggests that cattle may serve as a reservoir for MS IV strains, although our study does not provide evidence for the direction or likelihood of potential transmission between host species.

ST23 affects many host species (*12**,**27*). In humans, it is more commonly associated with carriage than with infection (*16**,**24*), although all ST23 in our study originated from infections. Most ST23 from humans have serotype Ia, and it has been suggested that serotype III originated in cattle, but the association between host and serotype is not absolute (*16**,**23**,**24*). In our collection, ST23 from humans was associated with MS Ia, whereas isolates from cattle were associated with MS Ia or MS III. PI profiles for ST23 matched those described for other countries (*11*). ST8, which was found across countries and species, and ST12, which was limited to Finland but isolated from both host species, have also been associated with carriage and invasive disease in humans, albeit at lower frequency than ST1 or ST23 (*16**,**24**,**25*). ST8 from both hosts had MS 1b and carried PI-1/2a, as previously described for isolates from humans (*11**,**28*). ST12 isolates from our study were associated with MS II, regardless of host species, and mostly carried PI-1/2a. This pattern matches reports of isolates from humans, although ST12 from humans may occasionally have serotype Ib (*11**,**28*).

The shared pathogen population may result from transmission between host species or shared exposure to an external source. Potential routes of transmission from cattle to humans include direct contact, exposure to cattle feces, and consumption of cows’ milk. Potential routes of transmission from humans to cattle include direct contact and indirect exposure to excreta from humans. In one prospective study, increased frequency of cattle exposure was associated with human colonization with *S. agalactiae*, although fecal colonization was detected in 1 cow only (*14*). The prevalence of fecal colonization on individual farms can be high (*26*), but gastrointestinal carriage is much more widespread among humans (*16**,**29*), which argues against exposure to cattle feces as a dominant reservoir for human colonization. Case reports and phenotyping of isolates show that raw milk consumption may lead to colonization of the human throat (*30*). This route may be of public health relevance in countries where milk is frequently consumed raw (e.g., Colombia) but not in countries where milk is routinely pasteurized (e.g., Europe and most of North America). In studies conducted in the United States, no significant association has been detected between consumption of milk and colonization of humans (*29**,**31*).

Several lines of evidence suggest that humans may be a source of infection for cattle. Experimental intramammary challenge of cattle with isolates of *S. agalactiae* from humans resulted in mastitis, although the duration of intramammary infection and hence the window of opportunity for transmission was less than that for infection with isolates from cattle (*32*). Similar observations have been made for naturally occurring bovine mastitis, whereby incidental cases in dairy herds were caused by strains that were otherwise predominantly found in humans (*33*). Epidemiologic studies also support the role of *S. agalactiae* sources other than cattle because introduction of the pathogen into dairy herds can often not be attributed to purchase of cattle, implying that alternative sources must exist (*9*). Some on-farm studies suggest that treatment of human oropharyngeal *S. agalactiae* carriers is a crucial step for eliminating *S. agalactiae* from dairy herds (*34*). Considering the frequent colonization of the human throat, gut, and urogenital tract with the shared STs observed in this study and the direct contact between human hands and the bovine mammary gland during milking, with or without use of gloves, a plausible mechanism for human-to-cattle transmission exists. The main difficulty in determining directionality of transmission between host species is establishing the order of events (i.e., which host species was positive first). Furthermore, efforts to detect alternative, potentially shared sources of *S. agalactiae* are limited by the preconceived but mistaken notion that *S. agalactiae* is an obligate intramammary pathogen in dairy cattle. Potentially shared sources include wastewater and surface water, including effluent from sewage treatment plants (*27**,**35**,**36*). Potential routes of within- and between-host species transmission, including horizontal and vertical transmission among humans (*12**,**31**,**37*) and contagious transmission among cattle via milking machines (*12**,**26*), are summarized in Technical Appendix Figure 6.

Alternatively, the co-occurrence of STs in both host species may not be the result of ongoing interspecies transmission but rather that of incidental spillover, with subsequent adaptation and dissemination within the new host, leading to parallel circulation of populations that have the same ST but encode host-specific adaptations elsewhere in the genome. This chain of events has been described for *Staphylococcus aureus* (*38*). Among *S. agalactiae* isolates from cattle, >90% are lactose fermenters, whereas the reverse is true among isolates from humans, providing an example of a host adaptation mechanism (*10**,**23*). The genes targeted by our PCR, *lacEFG*, form part of the Lac.2 operon, which is located on an integrative conjugative element and forms part of the *S. agalactiae* mobilome (*10*). Regulation of the *S. agalactiae* mobilome is a relatively new area of study (*39*); little is known about its contribution to host adaptation or interspecies transmission. Atypical combinations (i.e., *lacEFG*-positive isolates from humans and *lacEFG-*negative isolates from cattle) belonged to STs that are shared across host species, potentially indicating recent transmission events.

In our study, 2 STs occurred frequently but in only 1 host species. ST19 was commonly detected in humans but not in cattle. ST19 is generally rare in cattle, although its association with humans is not absolute (*11**,**12*). Conversely, ST103 was commonly found in cattle in our study and in Denmark, Norway, and China (*12**,**26**,**40*) but not in isolates from humans in our study or those mentioned in any of the references cited. ST103 and its SLVs were invariably associated with serotype Ia (*40*) and PI-2b (Technical Appendix Figure 5). We detected 4 new STs in the eBURST cluster around ST103, indicating ongoing evolution of this cattle-specific subpopulation. Those STs, and all other new STs detected in this study, were SLVs of known STs. The fact that all new STs were limited to 1 country and that they were SLVs of existing STs indicates that we still observe local microevolution but that we are starting to exhaust the variability in the *S. agalactiae* population.

In summary, according to MLST, molecular serotyping, and PI typing of contemporaneous *S. agalactiae* isolates from humans and cattle in Finland and Sweden, we identified 3 subpopulations: 1 from humans, 1 from cattle, and 1 from both hosts. The latter subpopulation accounted for more than half of the isolates, implying that the host species barrier separating *S. agalactiae* from both species may be more porous than previously thought. For STs commonly carried by humans (e.g., ST1 and ST23), the direction of transmission, if any, may be from humans to cattle. ST196/MS IV was relatively common among cattle, which may potentially constitute a reservoir of this recently recognized emerging pathogen of humans. The only characteristic that differentiated most isolates from the 2 species in this study was the ability to ferment lactose, which is encoded in the mobilome. Considering the new evidence for potential interspecies transmission of *S. agalactiae,* its emergence in adult humans and its reemergence in cattle, further studies into the mechanisms and frequency of transmission and host adaptation seem warranted.

Technical Appendix Origin, sequence type (ST), and molecular serotype (MS) of *S. agalactiae* isolates from dairy herds with multiple isolates available; origin and typing results for *S. agalactiae* isolates from humans and cattle; distribution of *S. agalactiae* isolates from humans; map of Sweden and Finland; distribution of STs of *S. agalactiae* isolates from humans; distribution of MSs across STs of *S. agalactiae* from humans and cattle*;* distribution of PI profiles across STs of *S. agalactiae* from humans and cattle.
