# Supplementary material for: Streptococcus agalactiae Serotype IV in Humans and Cattle, Northern Europe
Source: Emerg Infect Dis. 2016 Dec;22(12):2097–103. doi: 10.3201/eid2212.151447 (PMC5189126; doi:10.3201/eid2212.151447)
Supplement: Technical Appendix — Origin, sequence type (ST), and molecular serotype (MS) of S. agalactiae isolates from dairy herds with multiple isolates available; origin and typing results for S. agalactiae isolates from humans and cattle; distribution of S. agalactiae isolates from humans; map of Sweden and Finland; distribution of STs of S. agalactiae isolates from humans; distribution of MSs across STs of S. agalactiae from humans and cattle; distribution of PI profiles across STs of S. agalactiae from humans and cattle. [file 15-1447-Techapp-s1.pdf]

Article DOI: http://dx.doi.org/10.3201/eid2212.151447

Streptococcus agalactiae Serotype IV in Humans and Cattle, Northern Europe

Technical Appendix

Technical Appendix Table 1. Origin, sequence type and molecular serotype of bovine *Streptococcus agalactiae* isolates from dairy herds with multiple isolates available

Herd	Year	Sequence type	Molecular serotype	No. of isolates
2	2010	632	III	6
4	2010	103	Ia	2
5	2010	12	II	11
6	2010	1	V, inconclusive	13
				1
10	2011	103	Ia	2
11	2011	8	Ib	1
	2012	8	Ib	1
12	2011	183	V	2
16*	2011	635	V	1
	2012	1	V	1
18	2011	2	II	2

*Both isolates from this herd were included in herd-level analysis and comparisons between host species. For the remaining herds, a single representative isolate per herd was used. The two isolates from herd 16 were single locus variants of each other.

Technical Appendix Table 2. Origin and typing results for human and bovine *Streptococcus agalactiae* isolates, sorted by ST, MS, country, host species, year, and clinical origin

ST	MS	Country	Year	Host	Sex	Age (yrs)	Clinical origin	MRI ID	PI profile	PI1	PI2a	PI2b	lac	lacEFG
1	1a	FL	2012	H	F	31	carr	Z2–096	010	1	C	nd	0	0
1	II	FL	2012	H	F	27	carr	Z2–089	110	nd	B	nd	0	0
1	V	FL	2010	B	nd	nd	mast	Z2–026	110	nd	B	nd	1	1
1	V	FL	2011	B	nd	nd	mast	Z2–042	110	nd	B	nd	1	1
1	V	FL	2012	B	nd	nd	mast	Z2–057	110	nd	B	nd	1	1
1	V	FL	2012	B	nd	nd	mast	Z2–059	110	nd	B	nd	1	1
1	V	FL	2012	B	nd	nd	mast	Z2–062	110	nd	B	nd	1	1
1	V	FL	2012	B	nd	nd	mast	Z2–063	110	nd	B	nd	1	1
1	V	FL	2012	B	nd	nd	mast	Z2–066	110	nd	B	nd	1	1
1	V	FL	2012	B	nd	nd	mast	Z2–067	110	nd	B	nd	1	1
1	V	SE	nd	B	nd	nd	mast	Z2–141	110	nd	B	nd	1	1
1	V	SE	nd	B	nd	nd	mast	Z2–146	110	nd	B	nd	1	1
1	V	SE	nd	B	nd	nd	mast	Z2–154	110	nd	B	nd	1	1
1	V	SE	nd	B	nd	nd	mast	Z2–163	110	nd	B	nd	1	0
1	V	SE	nd	B	nd	nd	mast	Z2–164	110	nd	B	nd	1	0
1	V	SE	nd	B	nd	nd	mast	Z2–166	110	nd	B	nd	1	1
1	V	SE	nd	B	nd	nd	mast	Z2–167	110	nd	B	nd	1	0
1	V	SE	nd	B	nd	nd	mast	Z2–176	110	nd	B	nd	1	1
1	V	SE	nd	B	nd	nd	mast	Z2–177	110	nd	B	nd	1	1
1	V	SE	nd	B	nd	nd	mast	Z2–179	110	nd	B	nd	1	1
1	V	SE	nd	B	nd	nd	mast	Z2–183	110	nd	B	nd	1	1
1	V	FL	2011	H	F	57	UTI	Z2–069	110	nd	B	nd	0	0
1	V	FL	2011	H	F	89	UTI	Z2–117	110	nd	B	nd	0	0
1	V	FL	2011	H	F	83	UTI	Z2–118	110	nd	B	nd	1	0
1	V	FL	2012	H	M	0	SSTI	Z2–073	110	nd	B	nd	0	0
1	V	FL	2012	H	F	86	SSTI	Z2–100	110	nd	B	nd	1	1
1	V	FL	2012	H	F	31	UTI	Z2–070	110	nd	B	nd	0	0
1	V	FL	2012	H	F	65	UTI	Z2–072	110	nd	B	nd	0	0

ST	MS	Country	Year	Host	Sex	Age (yrs)	Clinical origin	MRI ID	PI profile	PI1	PI2a	PI2b	lac	lacEFG
1	V	FL	2012	H	F	60	UTI	Z2–074	110	nd	B	nd	0	0
1	V	FL	2012	H	F	68	UTI	Z2–075	110	nd	B	nd	0	0
1	V	FL	2012	H	F	33	UTI	Z2–076	110	nd	B	nd	0	0
1	V	FL	2012	H	M	73	UTI	Z2–091	110	nd	B	nd	0	0
1	V	FL	2012	H	F	64	UTI	Z2–097	110	nd	B	nd	0	0
1	V	FL	2012	H	F	90	UTI	Z2–114	110	nd	B	nd	0	0
1	V	FL	2012	H	F	82	UTI	Z2–136	110	nd	B	nd	0	0
1	V	FL	2012	H	F	32	carr	Z2–090	110	nd	B	nd	0	0
1	V	FL	2012	H	F	36	carr	Z2–119	110	nd	B	nd	0	0
1	V	FL	2012	H	F	26	carr	Z2–125	110	nd	B	nd	0	0
1	V	FL	2012	H	F	22	carr	Z2–131	110	nd	B	nd	0	0
1	VI	FL	2011	H	F	34	SSTI	Z2–115	110	nd	F	nd	0	0
1	VII	FL	2011	B	nd	nd	mast	Z2–046	100	nd	nd	nd	1	1
2	II	FL	2011	B	nd	nd	mast	Z2–054	010	1	A	nd	1	1
8	1b	FL	2011	B	nd	nd	mast	Z2–041	110	nd	B	nd	1	1
8	1b	FL	2011	B	nd	nd	mast	Z2–045	110	nd	B	nd	1	1
8	1b	FL	2011	B	nd	nd	mast	Z2–053	110	nd	B	nd	1	1
8	1b	FL	2012	H	F	27	SSTI	Z2–082	110	nd	B	nd	0	0
8	1b	FL	2012	H	F	49	UTI	Z2–137	110	nd	B	nd	0	0
8	1b	SE	nd	H	nd	nd	inv	Z2–187	110	nd	B	nd	0	0
8	1b	SE	nd	H	nd	nd	inv	Z2–189	110	nd	B	nd	0	0
10	0	SE	nd	B	nd	nd	mast	Z2–138	110	nd	C	nd	1	1
10	1b	FL	2012	B	nd	nd	mast	Z2–058	110	nd	B	nd	1	1
10	II	FL	2012	B	nd	nd	mast	Z2–068	110	nd	C	nd	1	1
10	II	SE	nd	B	nd	nd	mast	Z2–145	110	nd	C	nd	1	1
10	II	SE	nd	B	nd	nd	mast	Z2–161	110	nd	C	nd	1	1
12	II	FL	2010	B	nd	nd	mast	Z2–008	110	nd	A	nd	1	1
12	II	FL	2012	H	M	60	SSTI	Z2–079	110	nd	A	nd	0	0
12	II	FL	2012	H	F	51	SSTI	Z2–083	110	nd	A	nd	0	0
12	II	FL	2012	H	M	68	SSTI	Z2–094	010	0	E	nd	0	0
12	II	FL	2012	H	M	0	SSTI	Z2–105	110	nd	E	nd	0	0
12	II	FL	2012	H	F	51	SSTI	Z2–127	110	nd	A	nd	0	0
12	II	FL	2012	H	M	52	UTI	Z2–109	110	nd	A	nd	0	0
12	II	FL	2012	H	F	93	UTI	Z2–111	010	0	E	nd	0	0
12	II	FL	2012	H	F	67	UTI	Z2–133	110	nd	A	nd	0	0
12	II	FL	2012	H	F	32	carr	Z2–095	110	nd	A	nd	0	0
12	II	FL	2012	H	F	28	carr	Z2–134	110	nd	A	nd	0	0
17	III	FL	2012	H	F	77	UTI	Z2–093	101	nd	nd	m	0	0
17	III	FL	2012	H	F	60	UTI	Z2–121	101	nd	nd	m	0	0
17	III	FL	2012	H	F	35	carr	Z2–132	101	nd	nd	m	0	0
17	III	SE	nd	H	nd	nd	inv	Z2–185	101	nd	nd	m	0	0
19	II	FL	2012	H	M	76	UTI	Z2–078	110	nd	D	nd	0	0
19	III	FL	2011	H	F	38	UTI	Z2–116	110	nd	D	nd	0	0
19	III	FL	2012	H	F	82	SSTI	Z2–101	110	nd	D	nd	0	0
19	III	FL	2012	H	F	0	SSTI	Z2–102	110	nd	D	nd	0	0
19	III	FL	2012	H	F	94	UTI	Z2–088	110	nd	D	nd	0	0
19	III	FL	2012	H	F	79	UTI	Z2–110	110	nd	D	nd	0	0
19	III	FL	2012	H	F	13	UTI	Z2–112	110	nd	D	nd	0	0
19	III	FL	2012	H	F	13	UTI	Z2–113	110	nd	D	nd	0	0
19	III	FL	2012	H	F	84	UTI	Z2–122	110	nd	D	nd	0	0
19	III	FL	2012	H	F	83	UTI	Z2–123	110	nd	D	nd	0	0
19	III	FL	2012	H	F	58	UTI	Z2–124	110	nd	D	nd	0	0
19	III	FL	2012	H	F	25	carr	Z2–087	110	nd	D	nd	0	0
19	III	FL	2012	H	F	33	carr	Z2–106	110	nd	D	nd	0	0
19	III	FL	2012	H	F	26	carr	Z2–129	110	nd	D	nd	0	0
22	II	FL	2012	H	M	68	UTI	Z2–086	010	0	E	nd	0	0
23	nd	SE	nd	B	nd	nd	mast	Z2–162	110	nd	B	nd	1	0
23	1a	SE	nd	B	nd	nd	mast	Z2–158	010	1	C	nd	1	1
23	1a	SE	nd	B	nd	nd	mast	Z2–182	010	1	C	nd	1	1
23	1a	FL	2012	H	F	82	SSTI	Z2–092	010	1	C	nd	0	0
23	1a	FL	2012	H	M	87	SSTI	Z2–104	010	1	C	nd	0	0
23	1a	FL	2012	H	F	85	UTI	Z2–103	010	1	C	nd	0	0
23	1a	FL	2012	H	F	84	UTI	Z2–126	010	1	C	nd	0	0
23	1a	SE	nd	H	nd	nd	inv	Z2–190	010	1	C	nd	0	0
23	1a	SE	nd	H	nd	nd	inv	Z2–191	110	nd	C	nd	0	0
23	1a	SE	nd	H	nd	nd	inv	Z2–194	010	1	C	nd	0	0
23	III	SE	nd	B	nd	nd	mast	Z2–155	110	nd	F	nd	1	1
23	III	SE	nd	B	nd	nd	mast	Z2–160	110	nd	F	nd	1	1

ST	MS	Country	Year	Host	Sex	Age (yrs)	Clinical origin	MRI ID	PI profile	PI1	PI2a	PI2b	lac	lacEFG
27	III	FL	2012	H	F	29	UTI	Z2–080	010	0	D	nd	0	0
28	II	FL	2012	H	M	58	UTI	Z2–084	110	nd	D	nd	0	0
28	II	SE	nd	H	nd	nd	inv	Z2–186	110	nd	D	nd	0	0
28	II	SE	nd	H	nd	nd	inv	Z2–192	010	0	D	nd	0	0
103	1a	FL	2011	B	nd	nd	mast	Z2–044	001	0	nd	B	1	1
103	1a	FL	2011	B	nd	nd	mast	Z2–052	001	0	nd	B	1	1
103	1a	SE	nd	B	nd	nd	mast	Z2–142	001	0	nd	B	1	1
103	1a	SE	nd	B	nd	nd	mast	Z2–150	001	0	nd	B	1	1
103	1a	SE	nd	B	nd	nd	mast	Z2–153	001	0	nd	B	1	1
103	1a	SE	nd	B	nd	nd	mast	Z2–159	001	0	nd	B	1	1
103	1a	SE	nd	B	nd	nd	mast	Z2–165	001	0	nd	B	1	1
103	1a	SE	nd	B	nd	nd	mast	Z2–170	001	0	nd	B	1	1
103	1a	SE	nd	B	nd	nd	mast	Z2–171	001	0	nd	B	1	1
103	1a	SE	nd	B	nd	nd	mast	Z2–175	001	0	nd	B	1	1
144	1a	FL	2012	H	F	55	SSTI	Z2–107	010	1	C	nd	0	0
144	1a	FL	2012	H	M	30	UTI	Z2–071	010	1	C	nd	0	0
144	1a	FL	2012	H	M	77	UTI	Z2–085	010	1	C	nd	0	0
144	1a	FL	2012	H	M	35	UTI	Z2–108	010	1	C	nd	0	0
144	1a	FL	2012	H	F	26	carr	Z2–128	010	1	C	nd	0	0
144	1a	FL	2012	H	F	30	carr	Z2–135	010	1	C	nd	0	0
183	V	FL	2011	B	nd	nd	mast	Z2–049	110	nd	B	nd	1	1
196	IV	FL	2012	B	nd	nd	mast	Z2–060	110	nd	A	nd	1	1
196	IV	FL	2012	B	nd	nd	mast	Z2–065	110	nd	A	nd	1	1
196	IV	SE	nd	B	nd	nd	mast	Z2–140	110	nd	A	nd	1	1
196	IV	SE	nd	B	nd	nd	mast	Z2–148	110	nd	A	nd	1	1
196	IV	SE	nd	B	nd	nd	mast	Z2–149	110	nd	A	nd	1	1
196	IV	SE	nd	B	nd	nd	mast	Z2–169	110	nd	A	nd	1	1
196	IV	SE	nd	B	nd	nd	mast	Z2–172	110	nd	A	nd	1	0
196	IV	SE	nd	B	nd	nd	mast	Z2–180	110	nd	A	nd	1	1
196	IV	FL	2012	H	F	77	UTI	Z2–120	110	nd	A	nd	0	0
196	IV	FL	2012	H	F	32	carr	Z2–098	110	nd	A	nd	0	0
196	IV	FL	2012	H	F	30	carr	Z2–130	110	nd	A	nd	0	0
196	IV	SE	nd	H	nd	nd	inv	Z2–195	110	nd	A	nd	1	1
305	1a	FL	2012	H	M	53	SSTI	Z2–081	010	1	C	nd	0	0
314	1a	SE	nd	B	nd	nd	mast	Z2–144	001	1	nd	B	1	1
314	1a	SE	nd	B	nd	nd	mast	Z2–168	001	1	nd	B	1	1
314	1a	SE	nd	B	nd	nd	mast	Z2–173	001	1	nd	B	1	1
459	IV	FL	2012	H	F	27	carr	Z2–099	110	nd	A	nd	0	0
459	IV	SE	nd	H	nd	nd	inv	Z2–184	110	nd	A	nd	0	0
462	V	FL	2012	H	M	73	UTI	Z2–077	110	nd	B	nd	0	0
632	III	FL	2010	B	nd	nd	mast	Z2–001	001	0	nd	A	1	1
632	III	FL	2010	B	nd	nd	mast	Z2–002	001	0	nd	A	1	1
633	1a	FL	2011	B	nd	nd	mast	Z2–039	001	0	nd	B	1	1
633	1a	FL	2011	B	nd	nd	mast	Z2–050	001	0	nd	B	1	1
633	1a	FL	2012	B	nd	nd	mast	Z2–064	001	0	nd	B	1	1
634	V	FL	2011	B	nd	nd	mast	Z2–040	110	nd	B	nd	1	1
635	V	FL	2011	B	nd	nd	mast	Z2–047	110	nd	B	nd	1	1
636	V	FL	2011	B	nd	nd	mast	Z2–051	110	nd	B	nd	1	1
722	III	SE	nd	B	nd	nd	mast	Z2–139	110	nd	C	nd	1	1
723	III	SE	nd	B	nd	nd	mast	Z2–147	110	nd	B	nd	nd	1
724	1a	SE	nd	B	nd	nd	mast	Z2–143	101	nd	nd	m	1	1
725	IV	SE	nd	B	nd	nd	mast	Z2–152	110	nd	B	nd	1	1
726	IV	SE	nd	B	nd	nd	mast	Z2–156	110	nd	A	nd	1	1
726	IV	SE	nd	B	nd	nd	mast	Z2–178	110	nd	A	nd	1	1
726	IV	SE	nd	B	nd	nd	mast	Z2–181	110	nd	A	nd	1	1
727	1a	SE	nd	B	nd	nd	mast	Z2–157	001	1	nd	B	1	1
728	1a	SE	nd	B	nd	nd	mast	Z2–174	001	1	nd	B	1	1
751	II	SE	nd	H	nd	nd	inv	Z2–193	110	nd	D	nd	0	0
nd	1b	SE	nd	H	nd	nd	inv	Z2–188	110	nd	B	nd	0	0

*B, bovine; carr, carriage; F, female; FL, Finland; H, human; inv, invasive (sepsis or meningitis); M, male; MRI ID, Moredun Research Institute identification number; lac, lactose phenotype; lacEFG, *lacEFG*-PCR result; mast, mastitis; MRI ID, Moredun Research Institute identification number; MS, molecular serotype; nd, not determined; SE, Sweden; ST, sequence type; PI profile, Pilus island profile (in the order PI-1, PI-2a, PI-2b where 1 = positive and 0 = negative); PI-1 = occupation of integration site; PI-2a, adhesin gene (*gbs59*) allele; PI-2b, backbone protein (*san1519)* allele; SSTI, skin and soft tissue infection; UTI, urinary tract infection.

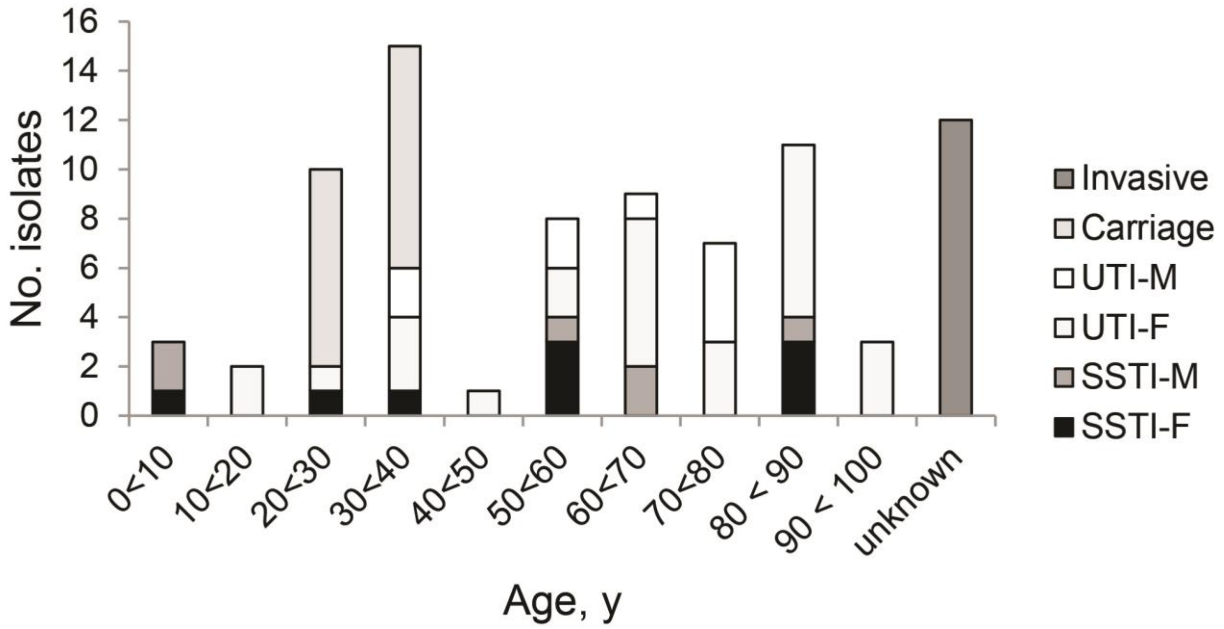

Technical Appendix Figure 1. Distribution of human *Streptococcus agalactiae* isolates across age groups and clinical origin. SSTI = skin and soft tissue infection; UTI = urinary tract infection; F = female; M = male.

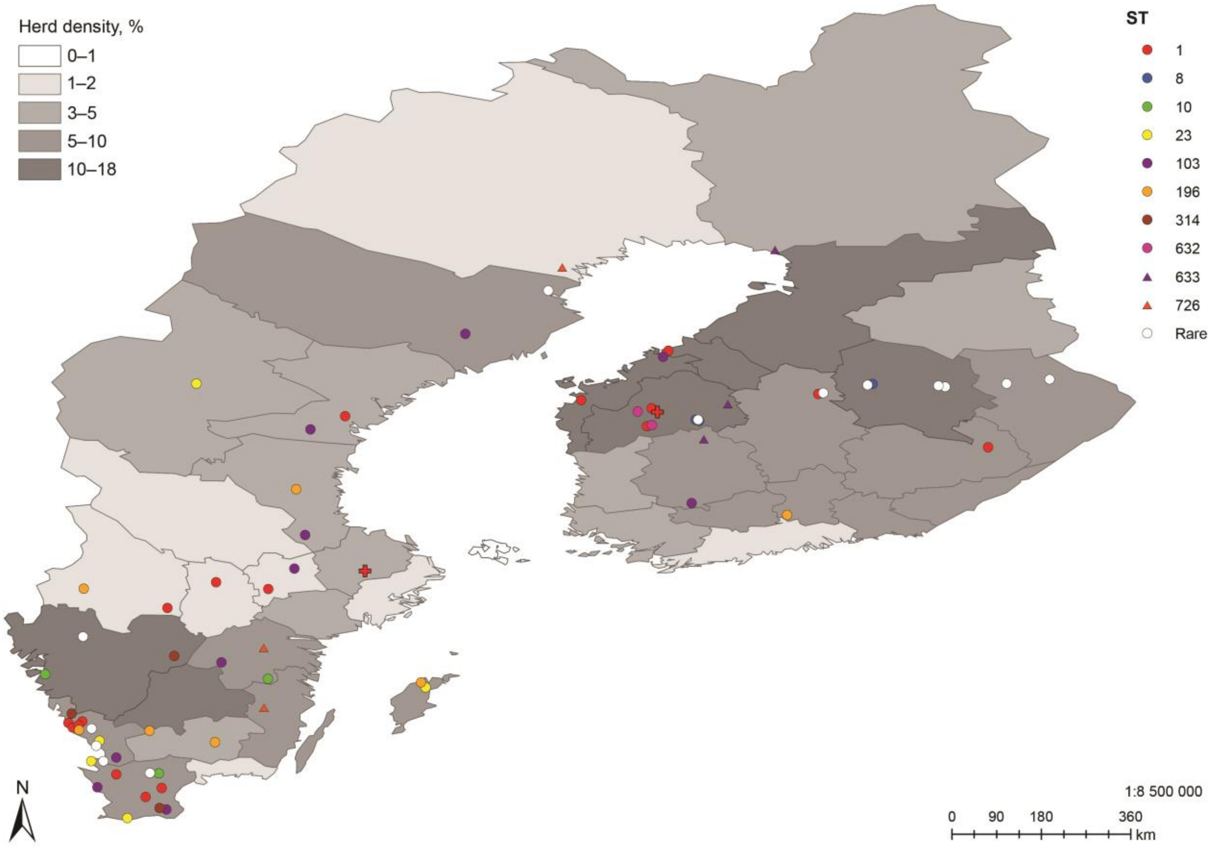

Technical Appendix Figure 2. Map of the study area (Sweden and Finland), showing the distribution on dairy farms (% of each country's dairy farms indicating by gray shading), location of hospitals (red crosses) and geographic origin of each sequence type (ST) identified among bovine isolates, with STs that were detected in more than one herd identified by circles of different colors or, for single locus variants of major STs, by triangles in the color of the major ST. The map was prepared in ArcMap 10.3.10 (Esri Inc., CA, USA). Location data were available for all Swedish herds (n = 45) and for 24 of 29 Finnish herds.

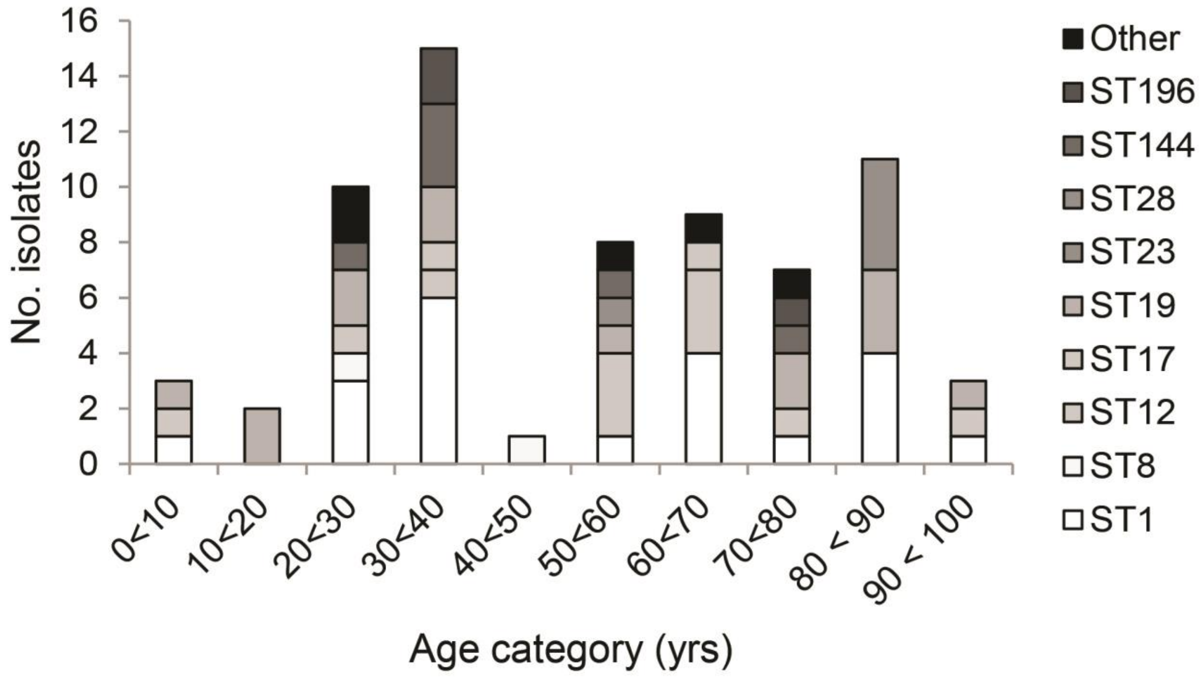

Technical Appendix Figure 3. Distribution of sequence types (ST) of human *Streptococcus agalactiae* isolates across age groups (A) and clinical origin (B). Age ranges shown with lowest age included, highest age excluded. SSTI = skin and soft tissue infection; UTI = urinary tract infection; F = female; M = male.

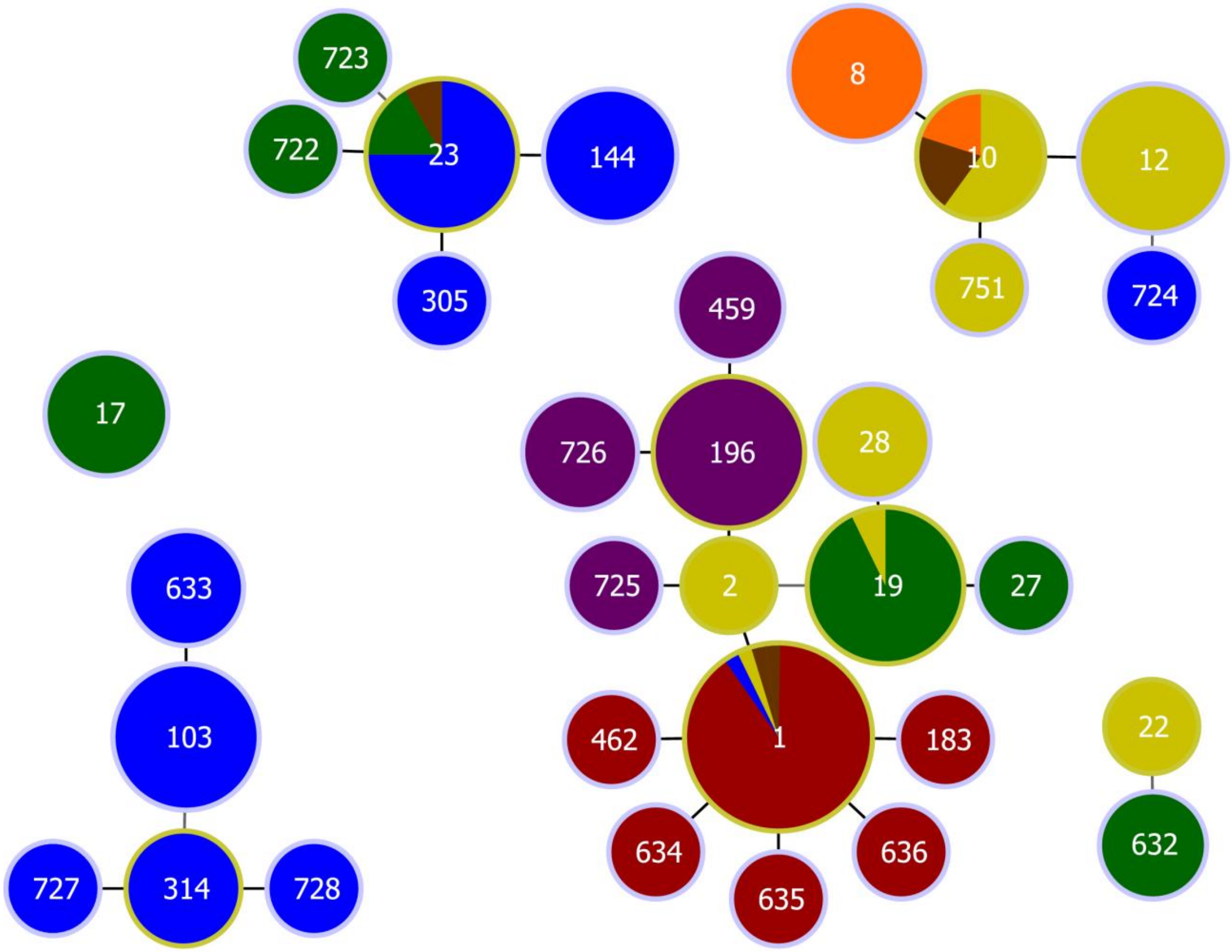

Technical Appendix Figure 4. Distribution of molecular serotypes (MS) across sequence types (ST) of human and bovine *Streptococcus agalactiae*. Clusters of STs were constructed using the goeBURST algorithm in PHYLOViZ (*1*) and include single locus variants and double locus variants connected by black lines. The size of the cluster and its colored segments is proportional to the number of isolates (logarithmically transformed). Blue = molecular serotype (MS) Ia; orange = MS-Ib; gold = MS-II, green = MS-III; purple = MS-IV; red = MS-V; brown = MS-VI, MS-VII or non-typeable. Numbers indicate STs.

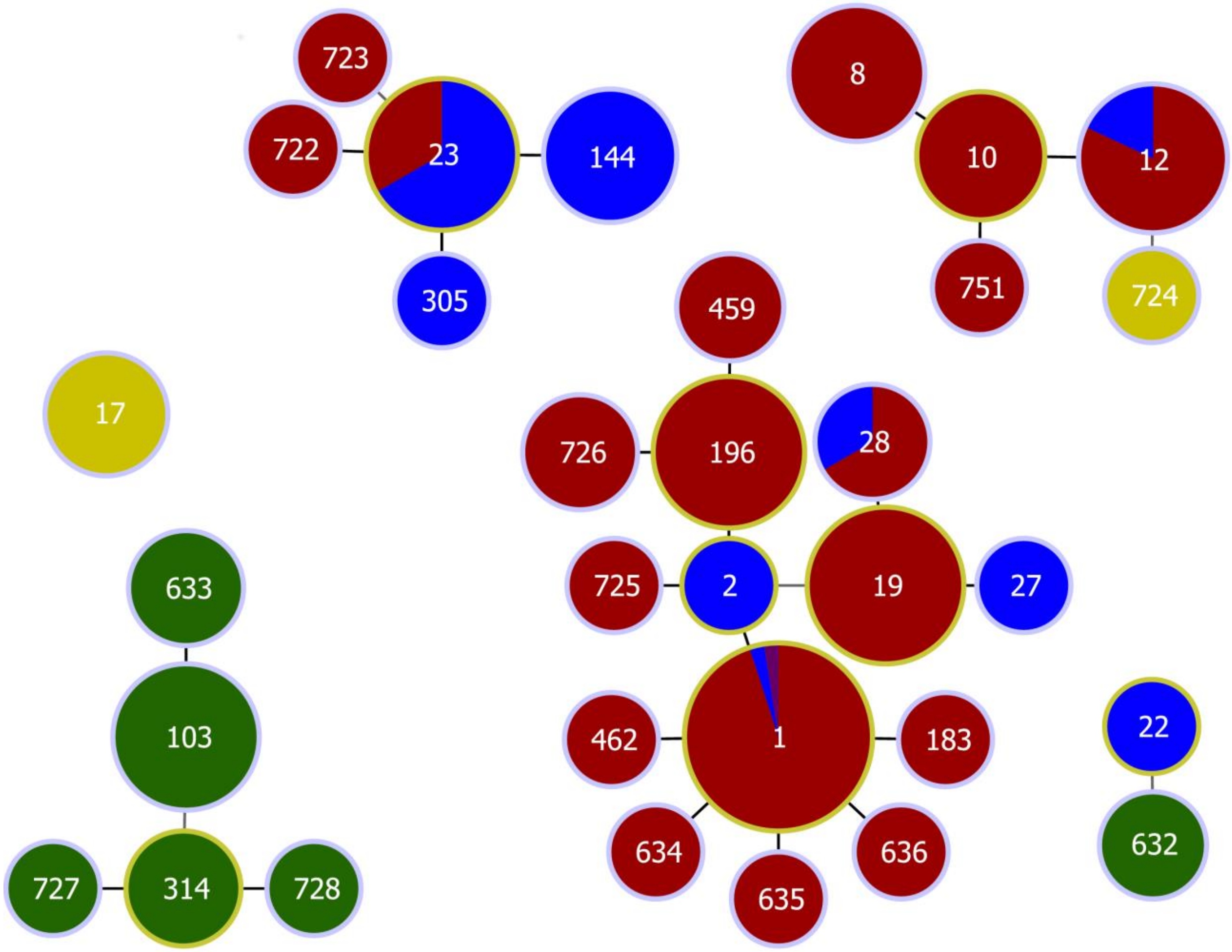

Technical Appendix Figure 5. Distribution of pilus island (PI) profiles across sequence types (ST) of human and bovine *Streptococcus agalactiae*. Clusters of STs were constructed using the goeBURST algorithm in PHYLOViZ (*1*) and include single locus variants and double locus variants connected by black lines. The size of the cluster and its colored segments is proportional to the number of isolates (logarithmically transformed). Red = PI-1/2a; Blue = PI-2a; Green = PI-2b; Gold = PI-1/2b; Purple = PI-1. Numbers indicate STs. The cluster around ST103 is bovine specific.

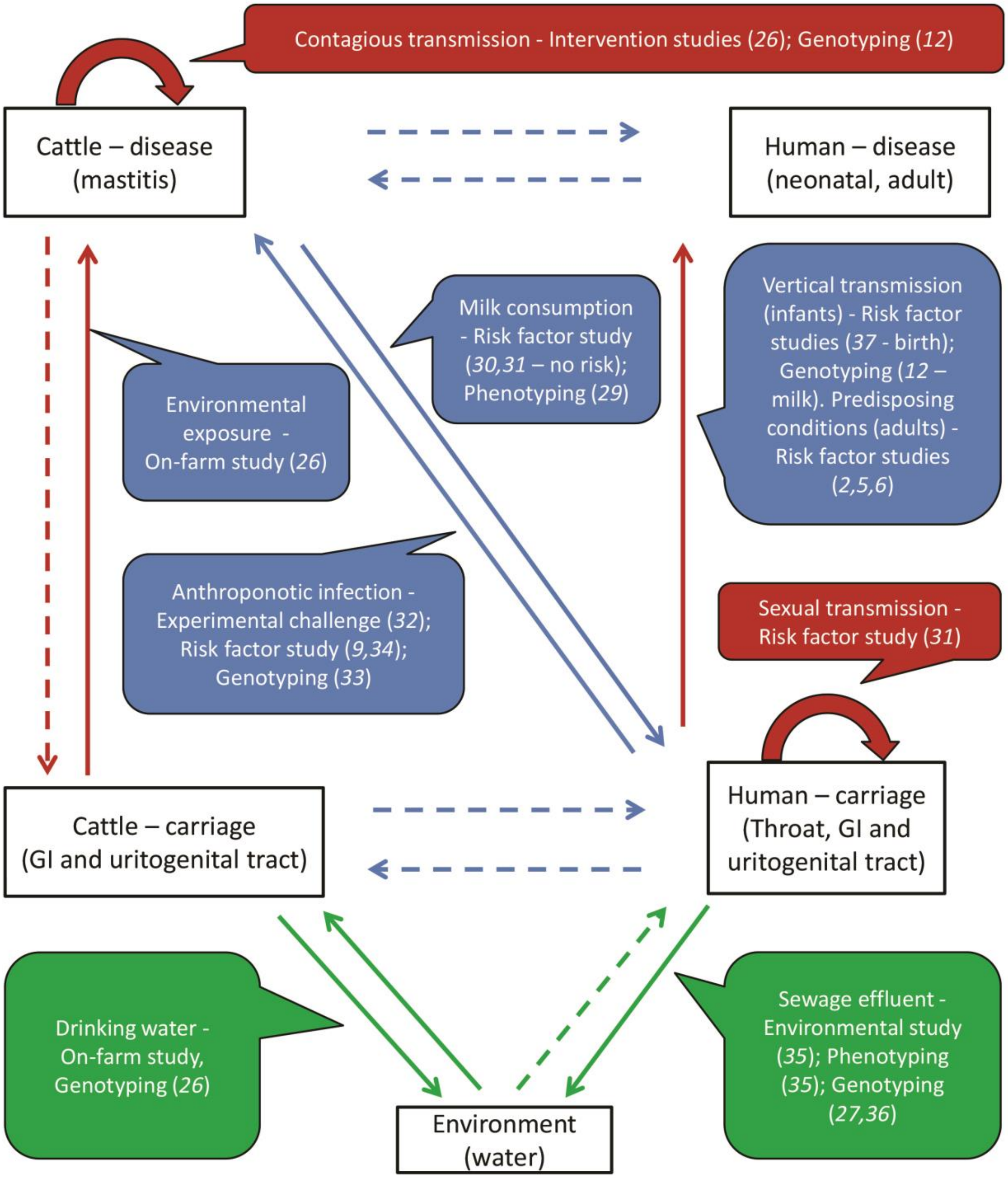

Technical Appendix Figure 6. Schematic representation of sources (boxes) and transmission routes (arrows) for *Streptococcus agalactiae* in humans, cattle and the environment. Full arrows are based on scientific evidence whereas dashed arrows were not supported by peer-reviewed publications at the time of writing. Call-outs list the mechanism of transmission and the supporting evidence. Intra-species transmission is shown in red, inter-species transmission in blue, and environmental transmission in green.

Reference

1. Francisco AP, Vaz C, Monteiro PT, Melo-Cristino J, Ramirez M, Carriço JA. PHYLOViZ: phylogenetic inference and data visualization for sequence based typing methods. BMC Bioinformatics. 2012;13:87. PubMed http://dx.doi.org/10.1186/1471-2105-13-87
